# Supplementary material for: Potential Use of Deep-Sea Sediment Bacteria for Oil Spill Biodegradation: A Laboratory Simulation
Source: Microorganisms. 2022 Aug 10;10(8):1616. doi: 10.3390/microorganisms10081616 (PMC9415916; doi:10.3390/microorganisms10081616)
Supplement: Supplementary file 1 [file microorganisms-10-01616-s001.zip › microorganisms-1826816-supplementary.pdf]

microorganisms

MDPI

Supplementary Files

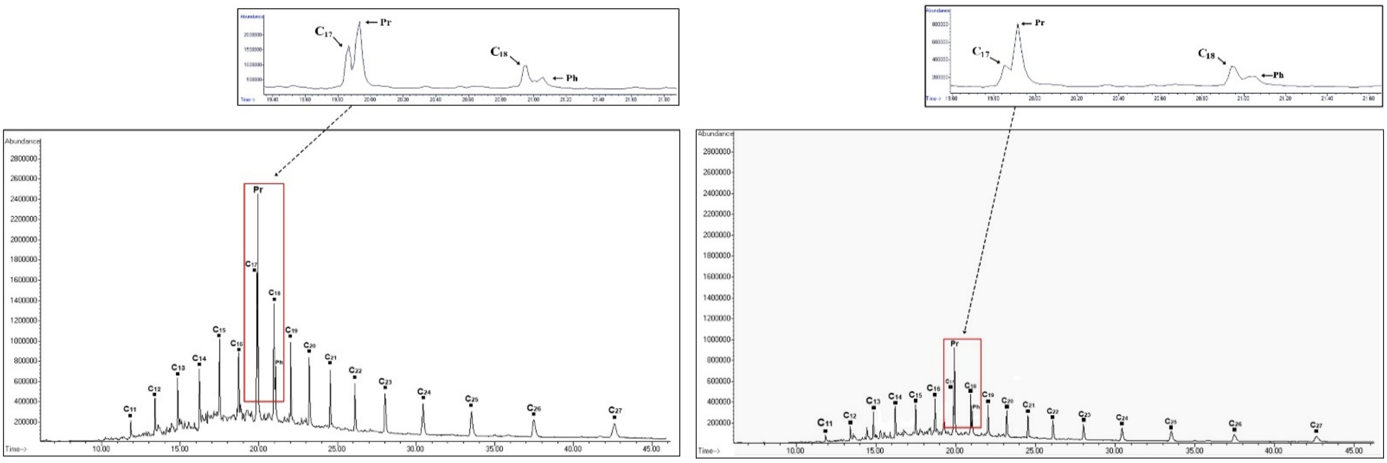

(**a**) (**b**)

Figure S1. Content change of paraffinic compounds found in crude oil: (**a**) before and (**b**) after biodegradation from GC-MS analysis.

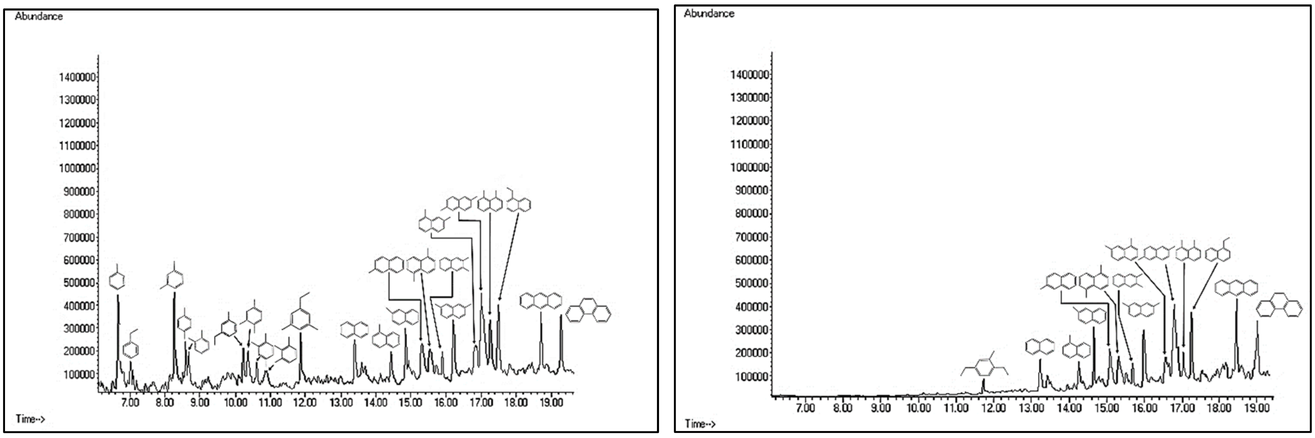

(a) (b)

Figure S2. Content change of aromatics compounds found in crude oil (**a**) before and (**b**) after biodegradation from mass spectra raw data of GC-MS analysis.
